# Development and Qualification of a Pseudotyped Virus‐Based Microneutralisation Assay for Influenza D Virus

**DOI:** 10.1111/irv.70245

**Published:** 2026-04-14

**Authors:** Maria Giovanna Marotta, Tobias Mapulanga, Meshach Maina, Janet Daly, Michele Camero, Pauline Mvan Diemen, Helen E. Everett, Emanuele Montomoli, Kelly da Costa, Nigel Temperton, Claudia Maria Trombetta

**Affiliations:** ^1^ Viral Pseudotype Unit Medway School of Pharmacy, Universities of Kent and Greenwich at Medway, Chatham Maritime Kent UK; ^2^ Department of Molecular and Developmental Medicine University of Siena Siena Italy; ^3^ Department of Life Sciences University of Siena Siena Italy; ^4^ One Virology, Wolfson Centre for Global Virus Research, School of Veterinary Medicine and Science University of Nottingham Nottingham UK; ^5^ Department of Veterinary Medicine University of Bari Aldo Moro Valenzano Bari Italy; ^6^ Virology Department Animal and Plant Health Agency Surrey UK; ^7^ VisMederi Srl Siena Italy

**Keywords:** accuracy, immunity, influenza D virus, linearity, precision, pseudotyped virus, qualification, robustness, specificity

## Abstract

**Background:**

Epidemiological surveillance of influenza D virus (IDV) has gained increased priority following recent serological findings indicating its potential zoonosis in humans. In this context, it is crucial to develop strong, reproducible, reliable and scalable immunological assays that can be quickly implemented in the surveillance of new emerging threats. Serology is a powerful tool for immune monitoring prior to infection and conducting epidemiological surveillance. However, the traditional microneutralisation (MN) assay requires wild‐type viruses, considerably limiting its accessibility for some laboratories. Pseudotyped viruses (PVs) allow for expanded usage since they are safer and more flexible for adaptation to specific strains and enable application in laboratories without implementation in high biosecurity containment.

**Methods:**

In this study, we conducted the qualification of a PV‐based MN (pMN) assay with IDV‐PVs that express the HEF glycoprotein of the D/Swine/Italy/199724‐3/2015 strain. The assay functionality was examined using 14 bovine serum samples, assessing key analytical parameters including accuracy, specificity, precision, linearity and robustness.

**Results:**

The findings demonstrate the IDV pMN assay to be an effective method for the detection of neutralising antibodies.

**Conclusions:**

Therefore, the assay can be a valuable tool to facilitate large‐scale surveillance and provide data to inform immunisation strategy development.

## 1 Background

Influenza D virus (IDV), a member of the Orthomyxoviridae family, was first isolated in 2011 from a nasal swab of a pig in Minnesota, United States (USA) [1]. Subsequently it has been identified in cattle, now recognised as its primary livestock host, as well as in small ruminants, horses, camels and dogs [2, 3, 4, 5, 6]. Although evidence of human exposure has been demonstrated through serological studies and virological surveillance, confirmed human to human transmission has not yet been demonstrated [7, 8, 9, 10]. Serological and molecular data suggest that the virus may pose a public health risk [11, 12]. IDV is typically transmitted between cattle through direct contact with aerosol droplets or contaminated fomites, causing mild clinical signs characterised by respiratory tract infections and signs of respiratory disease [13]. IDV is an enveloped, negative‐sense single‐stranded RNA virus with a segmented genome composed of seven segments, encoding for nine proteins: polymerases subunits PB1, PB2 and P3, the viral nucleoprotein (NP), the matrix proteins (M1 and M2), the non‐structural protein 1 (NS1), the nuclear export protein (NEP) and the single envelope glycoprotein, hemagglutinin esterase fusion (HEF) [9]. The HEF performs essential functions such as receptor binding, receptor destruction and membrane fusion by combining the activities of hemagglutinin (HA) and neuraminidase (NA) found in influenza A (IAV) and B viruses (IBV). This multifunctional protein is also a characteristic of the influenza C virus (ICV), with which the IDV shares approximately 50% nucleotide homology [14]. Phylogenetic analyses of the HEF glycoprotein have classified IDV into five lineages: D/660 and D/OK currently circulating in the Americas and Europe, D/Yama2016 and D/Yama2019 found circulating in Asia [2, 15, 16] and D/CA2019 recently reported in California, USA, after reassortment of the P3 gene from the D/OK lineage with the genome segments from the D/660 lineage [17]. The classification of IDV genotypes is therefore based primarily on the HEF gene phylogeny as this segment is the main determinant of antigenic and genetic diversity.

Due to its wide host range, global distribution and genetic similarity with the human ICV [18], IDV has become a topic of growing scientific interest. The zoonotic transmission of avian‐origin IAV H5N1 from bovine to human populations underscores the need to monitor potential risks posed by adapted bovine influenza viruses and the need to monitor IDV [19]. However, traditional serological assays often require the handling of infectious viral isolates, which may not be available to all laboratories. In addition, IDV does not produce a strong cytopathic effect (CPE) in cell culture, making traditional microneutralisation (MN) assay more laborious.

Pseudotyped viruses (PVs) offer a safe alternative for evaluating the humoral response to infection, especially when reagents are limited, as occurs with veterinary species. PVs are viral particles, unable to produce infectious progeny, that display the glycoproteins of interest, such as IDV HEF protein, in their native, functional form, on a structural core, allowing a direct and quantifiable evaluation of neutralising antibody responses in vitro [20, 21, 22]. While the use of PVs can reduce biosafety requirements and facilitate the implementation of testing in all laboratories, appropriate technical training remains essential to ensure reliable assay performance.

An effective serological assay must be accurate, capable of differentiating immune responses to various viruses and quantifying antibody levels over time and by different operators. In addition, the test should provide results proportional to the concentration of antibodies and demonstrate robustness under varying conditions and potential external factors.

In this study, we describe the qualification of a lentivirus PV‐based MN (pMN) assay using IDV‐PVs. This assay is designed to be fast, robust and convenient as well as serum‐ and antigen‐sparing [23]. Its performance has been evaluated by assessing key analytical parameters, such as accuracy, specificity, precision, linearity and robustness, in accordance with European Medicines Agency (EMA) guidelines [24], using bovine serum samples collected in the Apulia region, Italy. Although this report does not provide validation data, as defined by regulatory standards, it represents the development of analytical tools for wider application in IDV investigations. In this context, the use of pMN assay facilitates not only the monitoring of prior IDV infection but also the continuous surveillance of bovine respiratory disease (BRD), one of the most prevalent, deadly and costly diseases in cattle [25]. Considering the multifactorial nature of BRD and the role of IDV as a contributing pathogen, the consistent and reproducible measurement of the pMN assay to measure neutralising antibody responses may significantly contribute to mitigate the broader impact of BRD on cattle health and farm productivity, while supporting early actions to prevent or limit zoonotic threats.

## 2 Methods

### 2.1 Cell Line Maintenance

The human embryonic kidney 293T/17 (HEK293 T/17, ATCC: CRL‐11268a) cell line was used for transfection and PV generation. The swine testicular (ST, ATCC: CRL‐1746) cell line was used for transduction of IDV‐PVs and conducting assays. Both cell lines were cultured in complete medium Dulbecco's Modified Essential Medium (DMEM) (PANBiotech, P04‐04510), with high glucose and GlutaMAX supplemented with 10% (*v*/*v*) heat‐inactivated fetal bovine serum (PANBiotech, P30‐8500) and 1% (*v*/*v*) penicillin–streptomycin (10,000 units/mL of penicillin and 10,000 *μ*g/mL of streptomycin) (PenStrep) (Sigma, P4333) at 37°C and 5% CO_2_. Madin darby canine kidney (MDCK, ATCC: CCL‐34) cells, used to perform the conventional MN assay, were maintained at 37°C and 5% CO_2_ in Eagle's Minimum Essential Medium (EMEM) (Euroclone, Pero MI, Italy) supplemented with 10% (*v*/*v*) FBS (Euroclone, Pero MI, Italy), 1% (*v*/*v*) L‐glutamine (Euroclone, Pero MI, Italy), 1% (*v*/*v*) MEM Non‐Essential Amino Acids Solution 100X (NEAA) (Euroclone, Pero MI, Italy) and 1% (*v*/*v*) PenStrep (Euroclone, Pero MI, Italy).

### 2.2 Plasmid Production and Transformation

The HEF gene of IDV, strain D/Swine/Italy/199724‐3/2015 (GenBank accession number: KT592533.1), was synthesised by Thermo Fisher Scientific, UK, and subcloned into plasmid pI.18. The plasmid, together with the p8.91 HIV gag‐pol (gag‐pol expression plasmid), the luciferase reporter plasmid (pCSFLW) and plasmid pCAGGS encoding the human airway trypsin‐like (HAT) protease were employed for PV production [26, 27].

### 2.3 PV Production and Harvest

**FIGURE 1:**
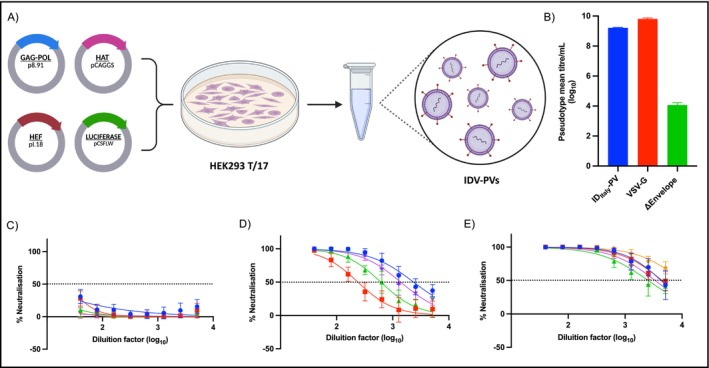
(A) Schematic representation of influenza D virus pseudotype virus (IDV‐PV) production. The production involves co‐transfection of the HEK293 T/17 cell line with four plasmids; plasmid p8.91 encoding the gag‐pol gene from HIV lentivirus for the viral core, firefly luciferase reporter plasmid pCSFLW, the pI.18 plasmid encoding the HEF glycoprotein and a HAT protease‐encoding plasmid to enhance maturation (created in https://BioRender.com). (B) Titration of IDV‐PVs. Target ST cells were transduced with IDV‐PVs, and infectivity was measured as relative luminescence units (RLU/mL). The observed titre is expressed in log_10_ and compared with the positive control vesicular stomatitis virus G glycoprotein‐expressing PV (VSV‐G PV) and the negative control (ΔEnvelope PV) not expressing a glycoprotein. (C), (D) and (E) Inhibition of IDV‐PVs by bovine samples with negative titre, low titre and high titre, respectively. Serum samples were tested using a starting dilution 1:40, with a dose input of PV suspension equal to 2 × 10^7^ RLU/mL as determined from titration. Each point of each graph observed represents the mean and standard deviation (SD) of three replicates per dilution performed by one operator. In each panel, coloured lines indicate individual serum samples within that category; colours are used solely to distinguish samples and do not encode additional information.

Plasmid transfection for IDV‐PVs production was performed in a 6‐well plate format as previously described [27] (Figure 1A). Briefly, 2 × 10^5^ cells/mL of HEK 293T/17 cells in complete DMEM were seeded 24 h before transfection and incubated at 37°C, 5% CO_2_ overnight. The next day, media was replaced, and cells were transfected with a plasmid DNA mix composed of 250 ng of HEF_Italy_ pI.18, 250 ng of gag‐pol p8.91, 375 ng of luciferase pCSFLW and 250 ng of HAT pCAGGS. All plasmid DNA were mixed in 100 *μ*L Opti‐MEM (Thermo Fisher Scientific 1985062, Paisley, UK) and 3.39 *μ*L FuGENE HD transfection reagent (Promega E2312 Madison, USA), added dropwise and followed by incubation for 15 min. The plasmid DNA‐OptiMEM mixture was then added to the cells with constant swirling. Forty‐eight hours post‐transfection, supernatants were collected, passed through a 0.45 *μ*m cellulose acetate filter and stored at −80°C prior to downstream use. PVs expressing the vesicular stomatitis virus G protein (VSV‐G) or the vector only not expressing a glycoprotein (ΔEnvelope) were used to create positive and negative control PVs, respectively.

### 2.4 PV Titration

Viral supernatants containing the expressed luciferase reporter protein were serially diluted twofold from 1:2 in a white 96‐well plate (Thermo Fisher Scientific 13610, UK) in duplicate. Target cells at a concentration of 1.75 × 10^4^ cells/well in 50 *μ*L were added per well. Cell‐only control wells were also included on each plate. Plates were incubated at 37°C, 5% CO_2_ for 48 h. After 48 h, the culture medium was removed and 25 *μ*L of Bright‐Glo (Promega, Madison, USA) luciferase assay substrate was added to each well and incubated for 5 min to lyse the cells and luciferase intensity was measured using the GloMax Navigator (Promega, UK) using the Promega GloMax Luminescence Quick‐Read protocol. Viral pseudotype titre was then determined in relative luminescence units/mL (RLU/mL).

### 2.5 Serum Samples

A total of 14 bovine serum samples were selected from a set of 246, collected in the Apulia region, Italy, all of which had been previously tested using a conventional cell‐based MN assay in house [28] and the wild‐type virus D/Swine/Italy/199724‐3/2015 also used to generate the PVs. Samples were grouped according to their MN titres, using conventional thresholds applied in influenza MN assay. Samples with titres < 10 were considered negative and indicated with a titre of 5, samples with titres ≤ 160 were classified as positive with low titre, and samples with titres ≥ 320 were defined as positive with high titre. From each category, samples were randomly selected to obtain a panel comprising five negative, four low‐titre and five high‐titre sera. Immunoglobulin‐depleted serum (Veterinary Lab Agency, PA0631) was used as a negative control. Hyperimmune serum generated by immunisation of a pig with D/Swine/Oklahoma/1334/2011 antigen was used as a positive control. All samples were heat‐inactivated for 30 min at 56°C before testing.

### 2.6 MN Assay

For the conventional MN assay [28], MDCK cells were seeded in 96‐well flat‐bottom plates and incubated for 4 h to allow adherence. Twofold serial dilutions of serum samples were prepared in UltraMDCK culture medium (Lonza, Walkersville, MD, USA) and then mixed with an equal volume of virus containing 100 TCID_50_/well of D/swine/Italy/199724–3/2015 viral strain. After 1 h of incubation at 37°C, 100 *μ*L of the serum–virus mixture was added to the MDCK cell suspension. Plates were incubated for 4 days and examined microscopically to assess CPE. The neutralising antibody titres were expressed as the highest serum dilution that prevented CPE in more than 50% of the wells.

### 2.7 pMN Assay

The pMN assay was performed in a white 96‐well plate format. Briefly, bovine serum samples were diluted in DMEM with starting dilutions of 1:40. Positive and negative control sera, included to validate each analytical run, were diluted starting from 1:100. PVs were added to each well at a concentration of 2 × 10^7^ RLU/mL. The serum‐PV mixture was incubated for 1 h at 37°C. After the incubation, 50 *μ*L of ST cells at a concentration of 1.75 × 10^4^ cells/well were added. The plates were then incubated at 37°C for 48 h, 5% CO_2_. After the incubation time, all media was removed and 25 *μ*L of Bright‐Glo Luciferase Assay System was added to each well. Following 5 min of incubation and cell lysis, the plates' luciferase intensity was measured using a GloMax Navigator luminometer with the Promega GloMax Luminescence Quick‐Read protocol. Viral pseudotype titre was then determined in RLU/mL.

### 2.8 Statistical Analysis

PV titres were determined using Microsoft Office Excel 365 (Version 16.92, Microsoft Corporation, Redmond, WA, USA). The PV titres obtained at each point in a range of dilution points were correlated to luciferase intensity expressed as RLU/mL, and the arithmetic mean was calculated. All statistical analysis was performed with GraphPad Prism Version 10.3.1 (GraphPad Software). Titres from pMN assays were first normalised for luciferase intensity based on cell only (100% neutralisation) and PV only (0% neutralisation) controls. IC_50_ values were calculated by a non‐linear regression model (log [inhibitor] vs. normalised response‐variable slope) [20]. For samples with undetectable neutralisation titre (IC_50_ = 0), an arbitrary value of log_10_ IC_50_ = 0.1 has been assigned for the graphical representation. An unpaired two‐tailed Student's test analysis was performed to calculate statistical significance (*, **, *** and **** = *p* < 0.05, significant differences; ns = not significant) [29].

### 2.9 Assay Accuracy, Specificity, Precision, Linearity and Robustness

The accuracy of the pMN assay was evaluated by measuring its sensitivity and specificity, using the MN assay as the reference method [30].

Sensitivity was calculated using the following formula:

$$\text{Sensitivity}=\left(\text{number of positive samples}/\text{number of positive samples}+\text{number of false negative samples}\right)\times 100$$

Specificity was calculated using the following formula:

$$\text{Specificity}=\left(\text{number of negative samples}/\text{number of negative samples}+\text{number of false positive samples}\right)\times 100$$

The specificity of the assay in differentiating the target analyte from non‐target analytes was also assessed by testing all samples against a heterologous PV strain of influenza A virus, A/red shoveler/Chile/C14653/2016 (H11N1), previously generated in the laboratory. A standard positive control for H11, chicken anti‐A/duck/Memphis/546/1974 (H11N9) serum (from OIE now WOAH) was also included to validate the analytical run.

The precision of an analytical procedure is defined by EMA in the ICH M10 guidelines as the closeness of repeated measurements under prescribed conditions [24]. Furthermore, precision was assessed at three levels: intermediate precision (including intra‐day and intra‐operator variation), repeatability and reproducibility.

Intermediate precision was analysed after two runs, performed by two operators, on two different days, with each sample tested in duplicate per run. Repeatability was assessed on six replicates performed on six different plates by the same operator.

Both intermediate precision and repeatability were determined by calculating the percent coefficient of variation (%CV), based on the geometric mean titres (GMTs) of IC_50_ titres (log_10_) and their standard deviation (SD) from the pMN assay. A CV ≤ 15% was considered acceptable [24]. Reproducibility was evaluated by conducting the assay by the Daly laboratory (University of Nottingham). The test was performed in duplicate by one operator, who prepared a new batch of the virus using the same protocol described above and employing the Steady‐Glo Luciferase Assay System (Promega, Madison, USA) as the reagent for readout analysis. Inter‐laboratory consistency was assessed using a Bland–Altman analysis performed on log_10_ IC_50_ values.

Linearity was evaluated by serial dilution of serum samples to demonstrate that the results were directly proportional to the amount of analyte. All samples were tested in duplicate by two different operators on two separate days. A twofold dilution scheme was applied, from a starting dilution of 1:40 to 1:5120. The GMTs were calculated for each dilution. A linear regression analysis was performed by plotting log_10_ serum dilution against log_10_ GMTs. The slope of the regression and the coefficient of determination (*R* ^2^) were used to assess linearity.

The robustness of the pMN assay was evaluated to assess its ability not to be affected by minor variations in method parameters. Standard conditions were defined as an incubation temperature of 37°C, a serum volume of 5 *μ*L and an incubation time of 48 h before evaluating luciferase activity. The following variations were tested: different incubation times (24, 48 and 72 h) applied to all serum samples; serum volumes (5 *μ*L ± 0.5 *μ*L); and incubation temperatures (37°C ± 0.5°C) applied to positive samples. Each condition was tested in duplicate by a single operator on the same day.

## 3 Results

IDV PVs were generated, and titres were compared with VSV‐G PV used as a positive control and ΔEnvelope PV used as a negative control. The results confirmed the high efficiency and robust titre of IDV‐PVs production [27] (Figure 1B). In the ΔEnvelope control, a low residual signal was observed, attributable to the formation of non‐infectious particles producing minimal reporter activity and the intrinsic background luminescence of the luciferase system. This residual signal was considered a cut‐off of the assay defining the minimum detectable infectivity.

A panel of 14 bovine serum samples was selected based on previously determined titres by MN assay, that were negative (titre = 5), low titre positive (titre ≤ 160) or high titre positive (titre ≥ 320). All 14 samples were tested using the pMN assay and IDV‐PVs. The negative titre sera remained below the neutralisation threshold of 50%, confirming the ability of the pMN assay in the identification of negative samples (Figure 1C). Low titre samples (Figure 1D) showed gradually decreasing neutralisation curves, consistent with lower antibody titres, while high titre samples (Figure 1E) demonstrated strong neutralising activity, as expected.

**FIGURE 2:**
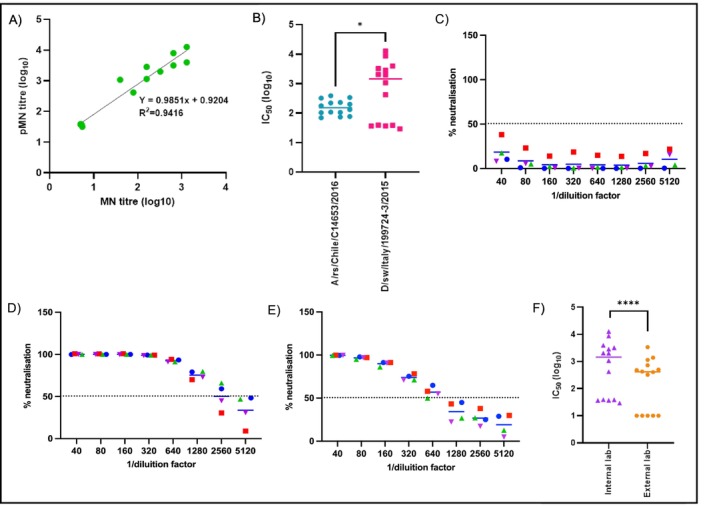
(A) Correlation of antibody titres detected by microneutralisation (MN) assay (*x*‐axis) versus pseudotyped virus based microneutralisation (pMN) assay (*y*‐axis). (B) Specifity. Specificity of the pMN was evaluated by testing all the 14 serum samples versus influenza A virus‐PV strain (A/red shoveler/Chile/C14653/2016). Statistical significance: *p* < 0.05 (*). (C), (D) and (E) Precision. Precision representation for all dilution factors in each serum group with (C) negative titre, (D) low titre and (E) high titre. Each sample was tested in duplicate, by two different operators and on two independent occasions. In each panel, coloured dots indicate individual serum samples within that category; colours are used solely to distinguish samples and do not encode additional information. (F) Reproducibility. All the serum samples were tested by a second operator concurrently in a separate laboratory.

To verify the accuracy of the pMN assay, neutralisation titres (log_10_) obtained from MN and pMN assays were used to directly compare the results (Table 1; Figure 2A). For all other qualification evaluations, titres of pMN were expressed as IC_50_ values.

**TABLE 1:** Comparison of neutralisation titres obtained using the microneutralisation (MN) assay with wild‐type virus and the pseudotyped virus‐based microneutralisation (pMN) assay.

Samples	MN titre (log_10_)	pMN titre (log_10_)
Negative titre		
169	0.70	1.58
171	0.70	1.59
184	0.70	1.56
194	0.70	1.56
196	0.70	1.50
Low titre		
188	2.20	3.45
4	1.90	2.62
193	1.60	3.03
185	2.20	3.06
High titre		
186	2.81	3.93
187	2.81	3.49
40	2.51	3.27
23	3.11	3.57
28	3.11	4.08

Of the 14 samples analysed, 9 were positive and 5 were negative; the results obtained in pMN assay were consistent with those obtained using the MN assay (Table 1). Only one sample, No. 188, classified in the low titre group according to the MN results, showed a higher titre in the pMN assay. The titres obtained were then used to assess the sensitivity and specificity of the pMN assay, both of which were determined to be 100% (Table 2). This good agreement between the two tests confirms the reliability of the pMN method for the detection of neutralising antibodies. The correlation of antibody titres detected by the MN assay and the pMN, through linear regression analysis (Figure 2A), demonstrates a strong linear relationship between the two methods, with a high correlation coefficient (*R* ^2^ = 0.9416). The regression equation (*Y* = 0.9851*x* + 0.9204) indicates near proportionality, as reflected by the slope of 0.9851, while the positive intercept (0.9204 log units) suggests that pMN titres tend to be slightly higher than those obtained in the MN assay. These results confirm the consistency of the pMN in the measurement of neutralising antibody titres.

**TABLE 2:** Sensitivity and specificity. Comparison of sensitivity and specificity values for the pseudotyped virus‐based microneutralisation (pMN) assay, using the microneutralisation (MN) assay as the reference method.

	MN	pMN
Negative	5	5
Positive	9	9
**Sensitivity**	100%	
**Specificity**	100%	

The specificity of pMN assay was also evaluated testing the same samples versus the IAV‐PVs strain A/red shoveler/Chile/C14653/2016 (H11N1) (Figure 2B) confirming that the pMN assay is specific. Indeed, all serum samples containing neutralising antibodies for D/Swine/Italy/199724‐3/2015 had high titres, whereas negative results were obtained versus H11N1, as observed with the negative group for IDV‐PVs. While some IC_50_ for H11N1 partially overlapped with the lower range of positivity for IDV likely due to cross‐reactivity at the lower limit of detection of the assay, overall, the IDV neutralising sera exhibited significantly higher titres (*p* < 0.05). These results confirm the assay's discriminatory capacity, showing significant differences between the two tested strains of influenza virus.

The precision parameter has been assessed by a detailed representation of all dilutions and sample groups (Figure 2C,D,E). Data demonstrate consistency of neutralisation responses, with low variability among the replicates, supporting assay agreement. The precision parameter was further evaluated by assessing the intermediate precision of the pMN assay. The GMTs of IC_50_ were compared under two conditions: same operator on different days (Table 3, intra‐operator variation) and same day but different operators (Table 4, intra‐day variation). In the intra‐operator variation (Table 3), a low CV was observed for the low titre group (1%), while the negative and high titre groups showed comparable CV values of 4% and 5%, respectively. Conversely, the intra‐day variation analysis produced a low CV (1%) for the high titre group, compared to 7% for the negative titre group and 4% for the low titre group. Despite these small differences, all %CV values remained within the acceptable threshold (≤ 15%) [24]. Regarding repeatability, the GMT of IC_50_ obtained from six independent repetitions performed by a single operator had a CV of 3% for all three groups, confirming the consistency of the assay (Table 5).

**TABLE 3:** Intra‐operator variation: same operator, different days. The table reports IC_50_ titres obtained by the same operator on different days, along with the corresponding standard deviation (SD) and coefficient of variation (CV).

	IC_50_ (log_10_)	SD	CV (≤ 15%)
Negative titre	1.45	0.05	4%
Low titre	3.03	0.02	1%
High titre	3.57	0.16	5%

**TABLE 4:** Intra‐day variation: same day, different operators. The table reports IC_50_ titres obtained by different operators on the same day, along with the corresponding standard deviation (SD) and coefficient of variation (CV).

	IC_50_ (log_10_)	SD	CV (≤ 15%)
Negative titre	1.45	0.02	7%
Low titre	3.03	0.14	4%
High titre	3.57	0.02	1%

**TABLE 5:** Repeatability. Repeatability results of the pseudotyped virus‐based microneutralisation (pMN) assay after six repetitions of one operator along with the corresponding standard deviation (SD) and coefficient of variation (CV).

	IC_50_ (log_10_)	SD	CV (≤ 15%)
Negative titre	1.38	0.04	3%
Low titre	2.78	0.08	3%
High titre	3.61	0.09	3%

Inter‐laboratory reproducibility was also assessed by comparing IC_50_ values obtained from analysis conducted by our laboratory (internal lab) and by a third‐party laboratory (external lab). Data from both laboratories have demonstrated a strong ability to discriminate between positive and negative samples, as indicated by a highly significant *p* < 0.0001 (Figure 2F). While the median IC_50_ values obtained by the internal laboratory were slightly higher than those reported by the external laboratory, and a greater variance in the data was observed, a Bland–Altman analysis confirmed the presence of a systematic inter‐laboratory difference. The external laboratory consistently reported lower IC_50_ values, with a mean bias of −0.56 log_10_ and limits of agreement ranging from −1.19 to +0.07 log_10_.

**FIGURE 3:**
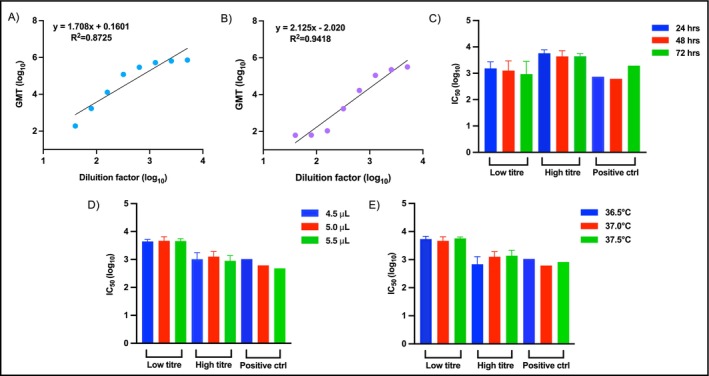
Linearity and robustness of the assay. Linearity representation for all dilution factors in (A) low titre and (B) high titre sample groups. Log_10_ of the dilution is indicated on the *x*‐axis and the log_10_ of the obtained geometric mean titres (GMTs) is reported on the *y*‐axis. Parameters of robustness that have been evaluated: (C) incubation time, (D) volume of serum samples and (E) temperature of incubation. Each bar indicates the IC_50_ ± standard deviation (SD) of two replicates per condition.

To evaluate the proportionality of the pMN assay response to serum concentration, linearity was assessed by linear regression analysis (Figure 3A low titre and Figure 3B high titre). The analysis yielded high *R* ^2^ values of 0.8725 for low titre samples and 0.9418 for high titre samples, indicating a good linear correlation and supporting the assay's capacity to provide reliable quantitative results across different antibody concentrations. The slopes obtained (1.708 for low titre samples, 2.125 for high titre samples) were outside the typical acceptance range for analytical methods (0.7–1.3) [31]. Despite the high gradients, no significant systematic deviations were detected in the residue analysis (data not shown), indicating that the test maintains proportional consistency and predictable behaviour across the concentration ranges tested.

The robustness of the pMN assay was assessed by testing all positive serum samples and the positive control under different experimental conditions. The impact of incubation time before readout was evaluated (Figure 3C) by comparing IC_50_ titres at different time points (24, 48 and 72 h). Titres obtained remained stable after 48 h of incubation (red bars), indicating this as the optimal condition for assay readout. While some variability was observed in the low titre samples after 72 h (green bar), likely due to the reduced neutralisation capacity, the high titre samples remained stable. The positive control also performed as expected, showing stability for 24 and 48 h, with a slight increase at 72 h attributable to the high neutralisation activity and prolonged incubation background signal. The impact of variations in serum volume and incubation temperature was then evaluated. Small deviations in serum volume (±0.5 *μ*L: 4.5, 5.0 and 5.5 *μ*L) were tested (Figure 3D). The IC_50_ values were consistent across all volumes, for low and high titre samples as well as the positive control, confirming the robustness of the analysis. Finally, fluctuations in the incubation temperature (±0.5°C: 36.5°C, 37.0°C and 37.5°C) were examined (Figure 3E), with consistent results observed under all conditions, supporting the robustness of the test compared to minor procedural variations.

## 4 Discussion

The suggested zoonotic potential of IDV represents a potential risk to human health. Evidence of human exposure is concerning and indicates that the virus has potentially acquired the ability to cross the species barrier from animals to humans [7, 8, 9, 10, 11, 32]. In addition to the public health implications, IDV also has the potential to impact animal health, the livestock industry and food safety. With the need to reduce antimicrobial use in food animals, monitoring pathogens that contribute to respiratory diseases becomes even more important. Overall, these considerations highlight the importance of effective surveillance tools to monitor spillover events and potential spread of IDV.

In this context, PVs are a valuable and safe alternative to measure neutralising antibody activity of samples without the need of wild‐type virus handling or when viral isolates are not available. During the SARS‐CoV‐2 pandemic, PVs were widely employed to assess the neutralising activity of vaccines and monoclonal antibodies [33]. They have also played an important role in detecting the rapid emergence of new viral variants carrying mutations on the spike protein, offering a safe model to assess immune evasion, accelerating vaccine development [34]. PVs are replication‐deficient viral particles that incorporate the enveloped glycoprotein of interest but lack the functional viral core components, remaining non‐infectious and able to mimic viral entry without the risks associated with wild‐type virus handling [22, 35, 36]. Adapting the protocol for lentiviral PV generation described by Ferrara and Temperton in 2018 [29], we developed a pMN assay for IDV. Following the assay development [27], here we present a qualification analysis to assess whether the assay is suitable for its intended purpose, thus demonstrating the assay performance in a comprehensive manner. While the performance of pMN assays has been well documented for other pathogens, such as Lassa fever virus and coronaviruses [31, 37, 38], to our knowledge this is the first study to address the validity of the IDV pMN assay. The availability of these already developed and qualified assays contributes to preparedness for novel disease incursions, allowing rapid and reliable serological assessments in case of future outbreaks in humans or veterinary species.

Evaluating key analytical parameters, we have provided statistical evidence that the IDV pMN assay ensures good performance relative to the existing conventional MN assay under different conditions and experimental settings. We included multiple animal samples with a different range of neutralising antibody titres when tested in the wild‐type virus MN, in order to consider the biological variability. The assay's accuracy, defined by sensitivity and specificity parameters, was confirmed with its strong concordance with the MN assay with wild‐type virus, in line with findings previously reported [30, 36, 39]. Sample No. 188 showed a higher neutralisation titre in the pMN assay. This difference may reflect the strong neutralising activity of this sample, together with the high sensitivity of the assay, attributed to the use of the luciferase reporter gene, which allows detection over a wide range of dilutions [29, 31]. In addition, the pMN assay also provides objective results as it is based on RLU measurements rather than subjective visual interpretation under a microscope [38, 40]. The specificity analysis confirmed that the IDV pMN assay effectively discriminated between IDV positive and negative samples. The limited overlap observed between the IDV low positive and negative titres and those obtained with IAV H11N1 may reflect the presence of conserved epitopes or shared structural motifs within HEF and HA glycoproteins. Although this overlap did not compromise the specificity of the assay, it evidences a potential limitation when testing samples from populations with a high prevalence of IAV. This threshold can be further evaluated in the future using serum panels containing larger numbers of test samples.

These observations support not only the accuracy of data but also the linearity of the analysis. Although the observed slope values were relatively high, the method showed good proportionality between analyte concentration and signal, confirming a linear relationship through dilution. This deviation reflects the inherent characteristics of bioanalytical assays using luciferase‐based detection systems, which can produce non‐linear responses at high antibody concentrations due to enzymatic saturation and biological variability in reporter gene expression, leading to steeper response curves [23, 41, 42]. In addition, a limited number of dilution points may have contributed to the slope differences. Furthermore, as assessed by Jansenn et al. [43], a slope statistically different from 1 does not automatically imply a biologically relevant deviation from proportionality. It is important to consider that the pMN assay relies on a variable slope logistic model to generate neutralisation curves, which naturally follow a sigmoidal pattern with a steep transition region. The HillSlope values obtained for high and low titre samples (absolute ranges approximately between 0.9–1.5 and 1.2–2.4, respectively) reflect this slope. Linearity can only be assessed within this transition region of the dose–sigmoidal response curve, which naturally produces linear regression slopes > 1. Overall, these considerations indicate that the high slopes observed in the linearity evaluation are consistent with the intrinsic behaviour of the pMN assay system.

An important aspect of an assay assessment is its ability to provide reliable results despite variability that may arise from differences in reagents, operators and cell line culture conditions. For this reason, the qualification analysis plays a key role in identifying and limiting the impact of such variability. In our case, the pMN assay proved high precision, confirming its ability not to be affected by changes that may occur in short periods of time such as days and operators. This was supported by the low %CV in evaluation of intra‐operator and intra‐day variations, highlighting its ability to provide consistent results across multiple analytical sessions. Since operator experience can affect the performance of the analyses, continuous monitoring over time is recommended to ensure long‐term consistency.

Inter‐laboratory selectivity in detecting differences under varying conditions was also evaluated. Minor differences were observed, in particular slightly lower IC_50_ values in the external laboratory results compared with the internal laboratory results. Statistical analysis confirmed a moderate but constant distortion between laboratories, likely due to the difference in the use of distinct luciferase detection reagents (Bright‐Glo vs. Steady‐Glo), which differ in luminescence intensity. Additional factors related to the complexity of sample handling steps such as shipping, freezing, thawing and processing may have further contributed to the observed variability [44]. However, the assay retained strong discriminatory power and produced stable results in both laboratories, indicating that this bias does not affect the overall assay interpretation. For future implementation in international surveillance networks, it will be important to enhance the resilience to procedural variation through harmonised protocols and shared reference materials. The development of standard operating procedures including reference antisera, internal controls and reagents would ensure greater consistency among laboratories.

We also undertook additional parameters that we used to evaluate the robustness. The results further confirmed reliability of the pMN assay demonstrating that it is resistant to potential variations commonly encountered during experimental procedures, such as minor pipetting inaccuracies or fluctuations in temperature of incubation.

Although the qualification was performed under conditions of limited serum volume and sample availability from a single species, these data suggest that the assay is suitable for its purpose. Future studies should aim to extend the assay evaluation by incorporating multicentric cohorts and to carry out a full validation based on the standardised and regulatory compliant procedures. Additionally, we will progressively improve the assay by incorporating glycoproteins from different IDV lineages, facilitating monitoring of emerging variants and their geographical distribution.

Taken together, our results demonstrate that the IDV pMN assay provides stable performance across different laboratory settings, supporting its applicability for wider use, particularly important in the context of global surveillance, where unified approaches are crucial. The consistency guaranteed by the qualification process is not simply a formal step, but necessary to ensure that data generated are robust, especially in critical domains such as surveillance of emerging infectious diseases, to improve prevention, early detection and response to health threats, including zoonotic diseases. With the ability to reliably measure responses to neutralising antibodies, the IDV pMN assay can allow effective coordinated monitoring, aligning global health preparedness for current and future zoonotic risks.

## Author Contributions

**Maria Giovanna Marotta:** conceptualization, methodology, formal analysis, data curation, investigation, visualisation, writing – original draft preparation. **Tobias Mapulanga:** investigation, writing – review and editing. **Meshach Maina:** investigation, writing – review and editing. **Janet Daly:** resources, writing – review and editing. **Michele Camero:** resources, writing – review and editing. **Pauline Mvan Diemen:** writing – review and editing. **Helen E. Everett:** writing – review and editing. **Emanuele Montomoli:** conceptualization, methodology, resources, funding acquisition, supervision, writing – review and editing. **Kelly da Costa:** conceptualization, methodology, supervision, writing – review and editing. **Nigel Temperton:** conceptualization, methodology, resources, supervision, writing – review and editing. **Claudia Maria Trombetta:** conceptualization, methodology, supervision, visualisation, writing – review and editing.

## Funding

This work was supported by the European‐Union, Next Generation EU, Tuscany Health Ecosystem, Spoke 7 (Grant Number CUP BC63C22000680007).

## Conflicts of Interest

Emanuele Montomoli is the founder and Chief Scientific Officer of VisMederi Srl (Italy). All other authors have no conflicts of interest to declare.

## Data Availability

The data that support the findings of this study are available from the corresponding author upon reasonable request.
